# Cattle-legislation in Austria

**Published:** 1896-06

**Authors:** 


Cattle-legislation in Austria. At the session of the Austrian Landtag, December 14, 1895, representatives von Proskowetz, Tausche, and colleagues addressed a communication to the Minister of the Interior, in which they referred to the late decline in prices in the Vienna cattle-market and in the markets of the other crown lands; to the condition of the Vienna cattle-market, and to the circumstance that, in spite of a marked decline in the price of cattle, the price of meat in Vienna had not been lowered. They then asked the following questions: 1. Does the minister intend to cause the report of the Agricultural Committee in regard to allowing the importation of cattle over the Roumanian boundary to become immediately the parliamentary order of the day? 2. Is the minister inclined to favor the enactment of regulations concerning the sale of meat in all lands, in order that this important article of food may be reduced to prices within the reach of the whole population?—*Ibid.*

